# Development and External Validation of a Home-based Risk Prediction Model of Natural Onset of Menopause—Teuta

**DOI:** 10.1210/clinem/dgae125

**Published:** 2024-03-05

**Authors:** Lum Kastrati, Pedro Marques Vidal, Klodian Dhana, Lia Bally, Irene Lambrinoudaki, Dion Groothof, Stephan J L Bakker, Michele F Eisenga, Taulant Muka

**Affiliations:** Institute of Social and Preventive Medicine, University of Bern, 3012 Bern, Switzerland; Graduate School for Health Sciences, University of Bern, 3012 Bern, Switzerland; Department of Diabetes, Endocrinology, Nutritional Medicine and Metabolism, Inselspital, Bern University Hospital, University of Bern, 3010 Bern, Switzerland; Department of Medicine, Internal Medicine, Lausanne University Hospital and University of Lausanne, 1005 Lausanne, Switzerland; Department of Internal Medicine, Rush University Medical Center, Chicago, IL 60612, USA; Department of Diabetes, Endocrinology, Nutritional Medicine and Metabolism, Inselspital, Bern University Hospital, University of Bern, 3010 Bern, Switzerland; National and Kapodistrian University of Athens, 2nd Department of Obstetrics and Gynecology, 15772 Athens, Greece; Division of Nephrology, Department of Internal Medicine, University Medical Center Groningen, University of Groningen, 9713 GZ Groningen, The Netherlands; Division of Nephrology, Department of Internal Medicine, University Medical Center Groningen, University of Groningen, 9713 GZ Groningen, The Netherlands; Division of Nephrology, Department of Internal Medicine, University Medical Center Groningen, University of Groningen, 9713 GZ Groningen, The Netherlands; Meta-Research Innovation Center at Stanford (METRICS), Stanford University, Stanford, CA 94303, USA; Epistudia, 3008 Bern, Switzerland

**Keywords:** menopause, prediction, validation, family planning

## Abstract

**Objective:**

To develop and externally validate a 10-year risk prediction model of natural onset of menopause using ready-to-use predictors.

**Design:**

Population-based prospective cohort study.

**Participants:**

Community-dwelling, premenopausal women aged 28 years and older enrolled in the Swiss (CoLaus) and Dutch (PREVEND) study.

**Main outcome measure:**

Incidence of self-reported natural menopause.

**Model development:**

Based on existing literature, 11 predictors were tested in this study. The CoLaus cohort was used to develop the model by applying the backward-elimination approach and Bayesian Model Averaging. Internal validation was performed by bootstrapping. External validation was performed using data from the PREVEND cohort and recalibrating the baseline survival estimate. C-statistics, calibration slopes, and expected/observed probabilities were calculated as measures of model internal and/or external performances.

**Results:**

The final analysis included 750 and 1032 premenopausal women from the CoLaus and the PREVEND cohorts, respectively. Among them, 445 (59%) from CoLaus and 387 (38%) from PREVEND experienced menopause over a median follow-up of 10.7 and 9 years, respectively. The final model included age, alcohol consumption, smoking status, education level, and systolic blood pressure. Upon external calibration in the PREVEND cohort, the model exhibited good discrimination, with a C-statistic of 0.888 and an expected/observed probability of 0.82.

**Conclusion:**

We present the first internally and externally validated prediction model of natural menopause onset using readily available predictors. Validation of our model to other populations is needed.

Menopause is an important event in a woman's life, as it marks the end of the reproductive lifespan. It can occur between the ages of 40 and 60 years, with the average age at natural menopause (ANM) for Western women ranging between 49 to 51 years (1). Menopause forecasts may be used to extrapolate the end of natural fertility, which typically occurs 10 years earlier (2). Being informed about the end of the female fertile lifespan would greatly assist in family planning and consideration of assisted fertility. This becomes particularly relevant due to the worrisome current trend of delaying childbirth with the increasing female participation in the workforce and the aging of the population (3). Projections report that the number of postmenopausal women will exceed 1 billion by the next decade (4). If menopause occurs earlier than expected, namely early menopause (eg, 45 or younger), it could lead to unforeseen infertility. Currently, as many as 10% of women experience early menopause (5).

Moreover, forecasting the ANM may also be beneficial for the tailored prevention and management of menopause-related risks and comorbidities. Studies have shown that women experiencing early menopause have a higher risk of developing chronic diseases such as type 2 diabetes, cardiovascular diseases, and osteoporosis. Moreover, these women live on average 3 years less as compared to women with normal ANM (5-8). On the other hand, late ANM (eg, 55 years or older) has been associated with a heightened risk of ovarian, breast, and endometrial cancers (9).

The feasibility of predicting the timing of ANM has been demonstrated in several studies that have utilized sex hormones and other biomarkers as predictive indicators, including family history and lifestyle factors as summarized in a recent systematic review (10). However, this systematic review of prediction models of ANM points out the methodological limitations of most of the models, in particular high risk of bias and lack of validation (10). Another pitfall is that the majority of these studies have developed prediction tools by using single or multiple hormone measurements, hindering the usability of these models in nonspecialist care and the cost-effectiveness from a health economic perspective.

Thus, because of the momentous impact that unforeseen infertility (also known as the “infertility trap”) might carry in times of worrisome declining birth rates, we aimed to develop a 10-year risk prediction model of the onset of natural menopause using data from 2 population-based cohorts, with readily available (sociodemographic, lifestyle, and medical history-based health information) factors related to ANM. [This timespan was chosen based on the availability of the data and corresponds to the mean follow-up time of the other prediction models of ANM (10).] This duration allows for a meaningful assessment of risk over a substantial timeframe while obtaining reliable estimates, allowing for timely discussion of conception and/or cardiometabolic prevention strategies.

## Methods

### Study Population

The model was developed using data from the CoLaus cohort and was externally validated in the PREVEND cohort. Detailed descriptions of the 2 cohorts can be found elsewhere (11, 12).

Briefly, the CoLaus cohort is a single-center population-based cohort of people of Caucasian origin living in Lausanne, Switzerland (11). With a follow-up period of every 5 years, we used the baseline assessment (2003-2006) and the second follow-up (2014-2017) of the CoLaus cohort. Inclusion criteria consisted of written informed consent and age 35 to 75. In the end, the baseline interview was completed by 2688 women. The study was approved by the Institutional Ethics Committee of the University of Lausanne, Switzerland.

The PREVEND study investigates the risk factors for and the prevalence and consequences of microalbuminuria in otherwise healthy adults (≥18 years) in the city of Groningen, the Netherlands (12). Briefly, all 85 421 inhabitants of the city of Groningen aged 28 to 75 years were invited, from 1997 to 1998, to participate in the study and were asked to complete a brief questionnaire and provide morning urine. The urinary albumin concentration (UAC) was determined in 40 856 responders. Pregnant women and participants with insulin-treated diabetes mellitus were excluded. Participants with a UAC ≥10 mg/L (n = 7768) were requested to participate in the cohort, of whom 6000 were enrolled. Additionally, a randomly chosen control group with a UAC of <10 mg/L (n = 3395) was invited, of whom 2592 were enrolled. These 8592 participants constitute the PREVEND cohort. A second screening round took place from 2001 to 2003, encompassing 6894 participants. We used data from the second screening (2001/2003) and the fourth follow-up (2009/2012) for external validation of the model. The PREVEND study has been approved by the local medical ethics committee (MEC 96/01/022) and was undertaken in accordance with the Declaration of Helsinki. All participants provided written informed consent. Patients or the public were not involved in the design, conduct, reporting, or dissemination plans of our research.

### Eligibility Criteria

Participants were excluded from the analysis if they (1) were males; (2) were postmenopausal at baseline or provided contradictory or no information on menopausal status; (3) had a hysterectomy and/or oophorectomy or polycystic ovarian syndrome (PCOS); (4) used hormone replacement therapy (HRT) at enrollment; (5) had missing data on independent variables; (6) were older than 55 years at baseline. Note that PCOS is associated with irregularities in menstruation, while HRT and hormonal contraception could restore bleeding in women, depending on how the progestin is prescribed. Thus, women with PCOS and using HRT were excluded to reduce the possibility of false-positive and false-negative cases, respectively.

### Data Collection

#### Assessment of the outcome/menopausal status and age

The outcome was assessed in the same way for all participants. Natural menopause was defined as self-reported natural cessation of menstruation. For the CoLaus cohort, menopausal status was assessed by asking the participants “Are you menopaused?” at baseline and “Do you still have your menses?” at the follow-ups.

In the PREVEND cohort, menopausal status was assessed by asking the participants the menstruation (“do you still menstruate”) question, which was answered as yes if they still menstruated, and, if not, time since the last menstruation was requested.

Participants who declared to have experienced menopause were asked to indicate ANM. In the CoLaus cohort, to correct for the recall bias, the reported ANM at the second follow-up was compared with the reported ANM at the first follow-up; if the difference was greater than 2 years, participants were excluded from the analyses (n = 37); otherwise, ANM was replaced with the average of the 2 reported ages. In the PREVEND cohort, information on ANM was categorical. To match the data in our development set, we averaged the age at menopause in each group and subsequently converted it to a continuous variable. For example, the category of ANM of 37 to 41 years was converted to 39.

In both cohorts, participants reported whether they had experienced natural or medically (surgical) induced menopause, as well as their history of using HRT. Additionally, in the CoLaus study, information on PCOS was collected.

#### Assessment of candidate predictors and other independent variables

Ready-to-use candidate predictors included in our analysis were chosen based on previous literature, biological plausibility, expert opinion, and availability in the respective cohorts (13-29). We identified 11 candidate predictors, summarized in Table 1.

**Table 1.** List of candidate predictors chosen from the literature

Candidate predictors	Studies that have shown a relation	No relation shown
Age	15-17	
Education	15, 18-21	22, 23
Smoking	15-18, 20-24	
Drinking-alcohol intake	13, 16	
Marital status	20, 22	18, 21
Body Mass Index	23, 25	15, 18, 20, 22, 26
Number of children/parity	15, 16, 18, 20, 23	21, 26
Oral contraceptives	18, 20	26
Age at menarche	15, 18	23, 27
Employment	15, 17-20	18, 22
Blood pressure	14	26

#### Predictors definition

The list of candidate predictors and how they were defined is described in Supplementary Table S1 (30). Continuous variables were kept as such, except body mass index (BMI), which was categorized to the literature standards for simplicity reasons should it be included in the final model (31). In the PREVEND cohort, only variables selected for the prediction model from the model development phase were assessed, and they were transformed to match CoLaus variables when needed.

### Sample Size

We were not aware of any previous prediction model on the topic that provided all the necessary parameters to perform a minimum sample-size calculation as suggested by Riley et al. Therefore, we used the 10 Events per Predictor Parameter rule of thumb as the minimum sample size for developing a prediction model (32).

### Statistical Analyses

#### Model development

STATA 17 and R Studio 4.3.2 were used for all analyses. Categorical variables were presented as numbers and proportions, while continuous variables were reported as medians and interquartile ranges or as average ± SD. Systolic blood pressure (SBP) was inversely transformed (1/SBP) to reach normal distribution, and diastolic blood pressure (DBP) was naturally log-transformed.

The risk prediction model was developed following the TRIPOD guidelines for model development and reporting (33). We performed multivariable Cox proportional hazards regression analyses to relate risk factors to the incidence of menopause and plotted the absolute risk. Follow-up of each participant began at the age of the baseline assessment and ended at the age of menopause, age of onset of HRT use (for participants who started HRT after enrolment), or end of the study period, which was until 2017. Mean follow-up time was calculated using the reverse Kaplan–Meier method.

All candidate predictors were tested for proportionality of the hazard assumptions based on the Schoenfeld residuals, and all predictors fulfilled the proportionality of hazard assumptions (34). We also checked for possible correlation between predictors by calculating the Spearman rank correlation coefficients (35). In case of high correlation, the correlated variables will explain the same variation in the outcome; therefore, only one of the correlated variables was kept. This is also a way of reducing the number of candidate predictors. Due to the high correlation between SBP and DBP (−0.83) and considering that SBP is used more often in prediction models of health outcomes, only SBP was kept for our analysis. We selected our model beginning with the defined set of candidate predictors (age, age at menarche, parity, use of contraceptives, alcohol use, level of education, BMI, smoking status, socioeconomic status, and SBP) using a backward selection procedure, considering the nonlinear relationship between continuous predictors and ANM. Fractional polynomials were used to identify the optimal functional form of continuous variables (using the STATA command fmp). We set a *P*-value of .1 so as not to be too stringent. Since only 6 participants had missing data on the development cohort, we performed a complete-case analysis; thus, no imputation method was used.

#### Model performance and internal validation

Calculation of the C-statistic and 95% confidence intervals (95% CI) was performed to evaluate the apparent discrimination performance of the model. The C-statistic is a measure of a model's discriminatory ability (ie, the ability to correctly classify individuals based on their outcomes), which can be interpreted as the probability that, for any randomly selected pair of individuals, the individual who experiences the event first has a higher predicted risk. We then calculated the calibration slope and plotted the linear predictor for the model in 4 risk groups to assess the separation across these groups (with better separation indicating good model performance). This is also indicative of the C-statistic. The risk groups were created using the specified centiles, which were defined by Cox's method (36). To assess for optimism generated in the model development process, calculations for any required shrinkage of the coefficients were made. We carried out internal validation to estimate optimism (overfitting level) and to correct measures of predictive performance (calibration slope) for model overfitting by bootstrapping 500 samples. The entire process of model selection was repeated in the bootstrap samples as per TRIPOD guidelines (see Supplementary Material, TRIPOD Checklist) (30, 33). We then applied each of these bootstrap sample models within the original dataset to estimate optimism in the performance statistics (difference in test performance and apparent bootstrap performance) of the C-statistic and calibration slope. To adjust for optimism after model development, we obtained estimates of a uniform shrinkage factor (the average calibration slope from each of the bootstrap samples) and multiplied these by the original β-coefficients to obtain optimism-adjusted coefficients (37, 38).

#### External validation

After model development and internal validation, we validated the model externally in the PREVEND cohort.

We fitted the calibration slope using the linear predictor as the only predictor in the model. The value of the calibration slope and the C-statistic for the discrimination of the model in the validation dataset were calculated. We then calculated the predicted survival probabilities for individuals at 10 years. Using the Kaplan–Meier estimate, the observed probability was obtained and the overall expected/observed ratio at 10 years was calculated. Next, we produced a calibration plot for the model In the external data. Subsequently, we recalibrated the baseline survival estimate of the model to account for miscalibration. Because external validation is done in populations with different characteristics (hence different baseline survival), we needed to recalculate the baseline hazard of the new dataset (39). We reestimated the baseline survival estimate and refigured the calibration plot to evaluate potential improvement in calibration. Calibration was presented as the ratio of observed to expected event probabilities and calibration plots to compare the observed vs predicted risks at 10 years.

#### Sensitivity analyses

Several sensitivity analyses were performed by (1) replacing SBP with DBP or keeping both variables to assess if this will have an impact on the model performance and (2) including antihypertensive therapy in the final model. First, we included antihypertensive therapy as a candidate predictor to check if backward elimination would choose antihypertensive treatment alongside SBP in the final model. Second, we excluded all the participants on antihypertensive medication (n = 38, 5%) to check if this would impact our results. To further assess the performance of the model, we computed different model performance measures across different thresholds. Given the absence of a presently established threshold for defining high-risk categories of menopause onset, we analyzed the distribution of predicted risks for developing menopause and computed values using the centiles proposed by Cox (36). We categorized women based on their predicted risk levels (those with a risk higher than 0.16, 0.5, or 0.84 to have experienced the event) and compared it to the true event rate in 3 separate analyses. For each centile threshold, we computed the area under the receiver operator curve, sensitivity, and specificity with the corresponding 95% CIs. We also computed the Brier's score to further assess the discrimination and calibration of our model. In addition, to ensure that the selected predictors would be supported by other model selection methods, we applied Bayesian Model Averaging (BMA) for survival analysis. The same 11 predictors were considered, and models created from all possible combinations of the candidate predictors were evaluated. Posterior probabilities (the probability that a predictor would be included in the model) were calculated, and predictors from the best 5 models were considered (R Package BMA). Finally, since categorizing continuous variables may cause loss of information, on a sensitivity analysis we explored the predictive utility of BMI as a continuous variable, both for the stepwise approach and the BMA approach (40).

## Results

A total of 750 and 1032 women fulfilled the inclusion criteria in the CoLaus and PREVEND cohorts, respectively. During a maximum follow-up period of 12.7 years and a median follow-up of 10.7 (CoLaus) and 9 years (PREVEND), 445 (59.3%) and 387 (37.8%) women, respectively, developed natural menopause at the end of the follow-up presented in Supplementary Figs. S1a, S1b, and S2a (30). General baseline characteristics of included participants are presented in Table 2.

**Table 2.** General baseline characteristics of all eligible participants from CoLaus and PREVEND cohorts

Characteristics	CoLaus (n = 750)	PREVEND (n = 1032)
Postmenopausal, n (%)	445 (59.3)	387 (37.5)
Early menopause, n (%*)*	14 (1.9)	52 (5.04)
Late menopause, n (%)	34 (4.5)	56 (5.4)
Age, years	42.5 ± 4.7	39.2 ± 6.04
Smoking, n (%)		
Never	348 (46.4)	369 (35.8)
Former	216 (28.8)	345 (33.4)
Current	186 (24.8)	318 (30.8)
Systolic blood pressure, mm Hg	114 (106.5-122)	115 (106-122)
Arterial hypertension, n (%)	85 (11.3)	37 (3.6)
Antihypertensive treatment, n (%)	38 (5.1)	37 (3.6)
Body mass index, kg/m^2^	22.83 (20.75-25.86)	**—**
Education, n (%)		
Low	192 (25.6)	241 (23.4)
Middle	227 (30.3)	646 (62.7)
High	331 (44.1)	139 (13.9)
Alcohol use, n (%)	531 (68.4)	778 (75.4)
Parity, n (%)	535 (71.3)	—
Birth control pills, n (%)	661 (88.1)	—
Age at menarche, years	13.2 ± 0.5	—
Living in couple, n (%)	485 (64.7)	—
Employed, n (%)	621 (82.8)	—

Data are means ± SD or median (interquartile range) or n (%) where indicated. Early (premature ovarian insufficiency < 40 years and early menopause 40-45 years) and late menopause (age at natural menopause > 55 years).

In CoLaus, hypertension was defined as systolic blood pressure ≥ 140 mm Hg and/or diastolic blood pressure ≥ 90 mm Hg and/or the use of antihypertensive medication.

In PREVEND, hypertension was defined systolic blood pressure ≥ 140 and/or diastolic blood pressure ≥ 90 mmHg (4), without a cardiovascular disease history and not using blood pressure-lowering agents.

In CoLaus, blood pressure was measured thrice on the left arm with an appropriately sized cuff, after at least a 10-minute rest in the seated position using an Omron® HEM-907 automated oscillometric sphygmomanometer, and the average of the last 2 measurements was used.

In PREVEND, blood pressure was calculated as the mean from 2 seated measurements using an automatic Dinamap XL Model 9300 series device.

In CoLaus, education was categorized into low (compulsory education, apprenticeship), medium (high school degree and secondary school), and high (university education).

In PREVEND, educational level was categorized into low (no, primary, basic vocational, and secondary education), middle (senior secondary vocational and general senior secondary education), and high (higher professional and higher academic education).

Only information on predictors that were included in the final model is available for the PREVEND cohort.

### Model Development and Apparent Performance

Out of 11 candidate predictors, 5 were included in the final model. Older age, alcohol abstinence, being a former or current smoker, having a lower level of education, and lower SBP were associated with a higher risk probability of developing menopause over the next 10 years. Fractional polynomial terms for the continuous predictors (age and SBP) were included in the final model to allow for nonlinear relations (Supplementary Table S2) (30). The model showed good apparent predictive performance (C-statistic of 0.837, 95% CI .819-.854) and perfect calibration (calibration slope = 1). This was confirmed as well by the good separation across the 4 risk groups, with a *P*-value of <.001, which also is indicative of the C-statistic (Supplementary Fig. S2b) (30).

### Model Internal Validation

Internal validation showed some overfitting. The calibration slope, which was previously perfect (calibration slope = 1), decreased to 0.949 after internal validation. The C-statistic did not significantly change (0.833 from 0.837 as it previously was). The estimates were multiplied by 0.949 to obtain optimism-adjusted β-coefficients (Supplementary Table S2) (30). The final model with adjusted coefficients is shown in the Supplementary Material Box S1 (30).

### Model External Validation

**Figure 1.**
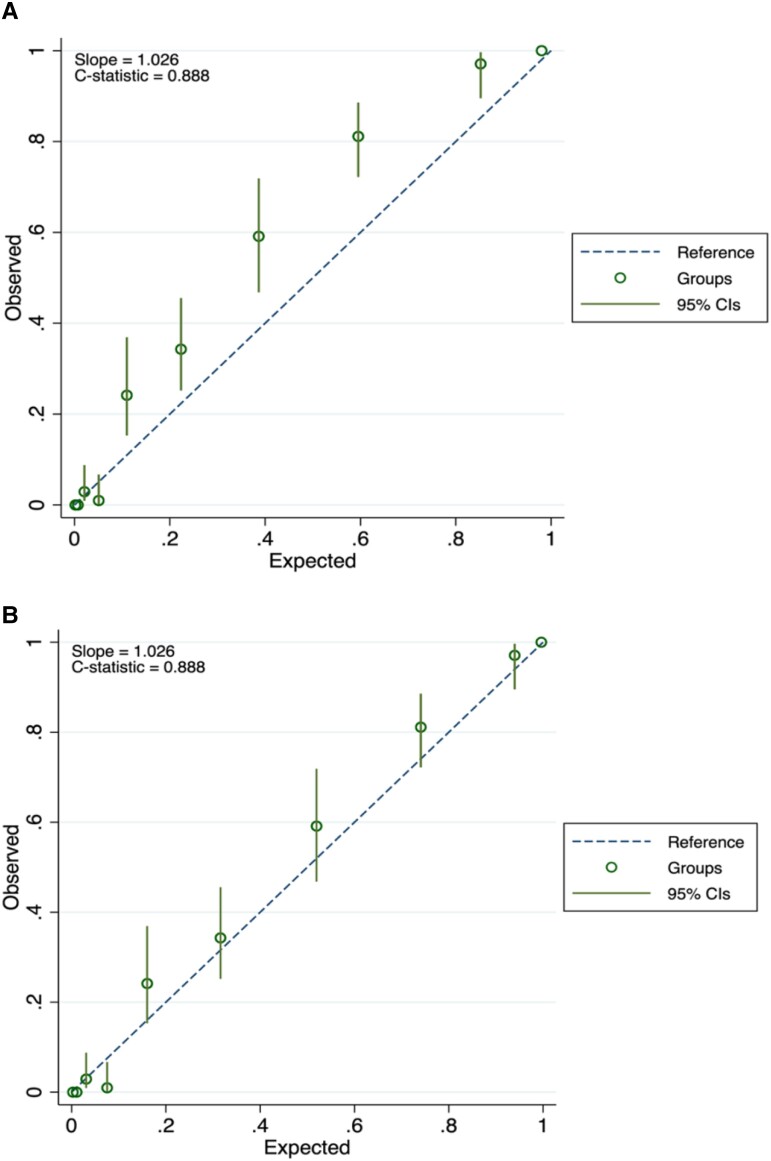
(A) Calibration plot in the external validation dataset. The calibration plot shows that the predictions are lower than the observed probabilities with most of the points lying above the reference line. (B) Calibration plot after external validation (recalibrating the intercept). Predictions now lie closer to the observed probabilities.

After fitting the final model in the validation dataset (PREVEND), we had a resulting C-statistic of 0.888 (95% CI .873-.900). The expected observed probability at 10 years (expected/observed) was 0.82, which shows that the model was underpredicting. The calibration slope was 1.026, confirming that the predictions were lower than the observed probabilities (Fig. 1A). Thus, we recalibrated the intercept and the baseline hazard. After recalibration, the baseline survival at 10 years was 0.321, instead of 0.470, which was before calibrating the model. After correcting for the systematic underprediction, the calibration slope showed better performance of the model (Fig. 1B).

### Sensitivity Analysis

Replacing SBP with DBP or using both predictors in the model did not change model performance. Including antihypertensive treatment in the group of candidate predictors did not impact the selected predictors, and excluding participants treated with antihypertensive treatment did not impact the results or the model performance. Our model showed good area under the receiver operator curve, sensitivity, and specificity across different thresholds [presented in Supplementary Table S3 (30)]. The model performed the best when a threshold of 0.5 was used, showing good sensitivity and excellent specificity. Using the risk threshold of 0.16 and 0.84, our model showed very high sensitivity and specificity, respectively. The model resulted in a Brier's score of 0.188, confirming good calibration and discrimination of our model. Using BMA for survival analysis for model selection resulted again in the same 5 predictors chosen by backward elimination. Finally, keeping BMI in its original continuous form did not change the results.

## Discussion

Using data from a Swiss population-based cohort, we developed an easy-to-use and cost-effective risk prediction model of natural menopause onset based on readily available predictors. Beginning with a set of predefined predictors chosen based on previous knowledge and expert opinion, our final model consisted of age, alcohol consumption, smoking status, level of education, and SBP. The model was selected using backward selection and Bayesian Model Averaging. Our model showed a good apparent performance, with a C-statistic of 0.837. We then externally validated our model in a Dutch population-based cohort, with similar participant clinical characteristics (Table 2). The expected/observed ratio of the model was 0.82, and the C-statistic 0.888. Before recalibration of the model, our model was underpredicting. After recalibrating the intercept to adjust for optimism, the new calibration slope showed better performance of the model. Our model performed well across different thresholds, suggesting that we can use different thresholds depending on the aim. Employing a lower threshold would allow us to lower the rate of false-negative cases whereas a higher threshold would allow lowering the rate of false positives.

### Our Model in Context With the Literature

To our knowledge, this is the first study to develop a risk prediction model of menopause onset relying only on readily available predictors and to externally validate it. This is in contrast with another prediction model that used readily available predictors and did not perform any form of validation (41).

While there was a study that performed external validation (cross-validation), the corresponding prediction models relied on single or multiple hormone measurements (42).

The C-statistics of the previously published ANM prediction models ranged from 0.71 to a maximum of 0.95. However, all these models resulted in high risk of bias for at least 2 domains when assessed with the PROBAST tool (10).

The included predictors in our final model are in line with previous findings, being constantly reported as factors associated with the timing of menopause. A meta-analysis by Taneri et al found a protective effect of alcohol consumption in relation to ANM. Low to moderate alcohol amounts were linked to a later onset of menopause. In contrast, increased amounts of alcohol consumption, also known as binge drinking, have not shown to play a role in the timing of menopause. Even though the underlying mechanisms remain unknown, it is postulated that alcohol consumption might influence hormone levels. While studies exploring the association between alcohol consumption and FSH levels show contradictory findings, there was a consistent estradiol-raising effect of low to moderate alcohol consumption demonstrated. On the other hand, alcohol consumption could also serve as a representation of certain lifestyle habits, including diet and physical activity (13). Smoking is a well-known risk factor of earlier ANM. It causes irreversible damage to the ovaries by increasing the rate of apoptosis of oocytes, which also explains our findings on former smokers having a higher risk than never-smokers (43). Moreover, smoking was also associated with decreased values of anti-Müllerian hormone—values that linked to earlier ANM (44). In line with our research, not only current but also former smoking increases the risk of experiencing an earlier menopause (28). The literature has been divided when it comes to the relation of menopause onset and level of education. However, most of the literature supports the hypothesis that more educated women have a later onset of menopause (45). Lastly, in line with our findings, a Mendelian-randomization study showed that women with higher blood pressure have a later onset of menopause (14). No association was found between BMI and ANM. This goes in line with the growing body of literature on negative findings between BMI and ANM (Table 1).

### Strengths and Limitations

Our study contained a high number of participants experiencing the event of interest, with almost 60% of the study sample having experienced the outcome during the follow-up. The high event rate allowed us to obtain reliable estimates of the calibration and performance of our model (since only events and not the total number of participants contribute to the log partial likelihood of a Cox regression). Moreover, both cohorts have a population-based prospective design. The wide age range increases the possibility of capturing women experiencing early menopause. The readily available predictors permit a cost-effective use of this prediction model within routine care and on a population level, utilizing the risk calculator provided at epistudia.com.

Nevertheless, some limitations need to be considered. Both studies used self-reported status of menopause and menopausal age, making it prone to recall bias. Moreover, in the PREVEND cohort, ANM was categorized, losing information on the outcome. Although there might be a potential for misclassifying menopause based on how it was identified (distinguishing between menopause- and nonmenopausal-related amenorrhea), we mitigated this by excluding all women who declared they were postmenopausal at the baseline, reducing the likelihood of such misclassification. Moreover, no women who declared they were on hormonal contraception declared they had amenorrhea. Having the low number of participants experiencing early and late menopause, limits the predictive power of our model in those populations. The mean baseline age in the development cohort was 42.5 ± 4.7 years, limiting the generalizability to younger populations. However, both the developing and the validating cohorts contain a wide range of age, including women in their early 30s. Moreover, predictors used in our model have been consistently validated in younger populations as well (46, 47). The PREVEND cohort used for external validation also had slightly different inclusion criteria (microalbuminuria in otherwise healthy subjects). Participants with microalbuminuria in PREVEND did not show differences in smoking status but were older and had higher blood pressure compared to participants without microalbuminuria. This, alongside the younger baseline age and different outcome incidence as compared to CoLaus, could also partially explain why our prediction model was underpredicting in the PREVEND cohort (48). Lastly, both CoLaus and PREVEND consist of Western populations. However, ANM varies between different populations; therefore, we should be reserved when drawing conclusions on the generalizability of our findings (9).

### Implications of our Findings and Future Prospects

We provide an easy-to-use risk prediction model of ANM as a supportive tool for family planning. This prediction model can help women who plan on having children in the future predict their ANM, aiding counseling for the possibilities that assisted reproductive technologies offer. This model can have an impact on the concerning decline in birth rates by providing information relatively early in life about the fertility lifespan and avoid the infertility trap. In addition to the scope of use in terms of family planning, this prediction model can be used for timely discussion and consideration of HRT and/or preventive strategies against menopause-related risks and comorbidities. The latter is particularly relevant for women who are already at risk or have established cardiometabolic or bone diseases, or cancer. Our findings highlight that even former smokers have a higher risk for earlier menopause as opposed to never-smokers, suggesting irreversible damage on the function of the reproductive tract of women. This study also shows an ANM delaying effect of alcohol consumption; however, no safe doses of alcohol consumption have been established, therefore our findings should not be taken as recommendations for alcohol consumption (49). To illustrate its utility in clinical practice, the final model and some worked examples can be found in Box S1 and S2 in the Supplementary Materials (30).

Efforts should be made to validate this model in other European and non-European populations. There are also possibilities to improve this model with other easily accessible predictors or standard hormone assays. As an example, mothers’ ANM has been proven to be a strong predictor of daughters’ ANM, information lacking in our cohorts. Moreover, further research should be done on other factors such as omics or genetic risk scores for a more personalized approach. Methodological limitations of currently available prediction models underscore the need for methodological rigor, exploring and validating the true potential of hormonal and nonhormonal models to predict time to menopause.

## Conclusion

We provide the first internally and externally validated risk prediction model of natural menopause onset consisting of age, smoking status, educational level, alcohol consumption, and systolic blood pressure—all readily available predictors. Validation in other populations and adapted variable selection may further increase its clinical utility and predictive capacity in the future.

## Data Availability

The data of the CoLaus|PsyCoLaus study used in this article cannot be fully shared as they contain potentially sensitive personal information on participants. According to the Ethics Committee for Research of the Canton of Vaud, sharing these data would be a violation of the Swiss legislation with respect to privacy protection. However, coded individual-level data that do not allow researchers to identify participants are available upon request to researchers who meet the criteria for data sharing of the CoLaus|PsyCoLaus Datacenter (CHUV, Lausanne, Switzerland). Any researcher affiliated with a public or private research institution who complies with the CoLaus|PsyCoLaus standards can submit a research application to research.colaus@chuv.ch or research.psycolaus@chuv.ch. Proposals requiring baseline data only will be evaluated by the baseline (local) Scientific Committee (SC) of the CoLaus and PsyCoLaus studies. Proposals requiring follow-up data will be evaluated by the follow-up (multicentric) SC of the CoLaus|PsyCoLaus cohort study. Detailed instructions for gaining access to the CoLaus|PsyCoLaus data used in this study are available at www.colaus-psycolaus.ch/professionals/how-to-collaborate/. The data underlying the results presented in this study can be made available by the data manager of the PREVEND study, Ms. Lyanne Kieneker, l.m.kieneker@umcg.nl. Public sharing of individual participant data was not included in the informed consent form of the study, but data can be made available to interested researchers upon reasonable request.
